# Comparison of conventional and advanced *in vitro* models in the toxicity testing of nanoparticles

**DOI:** 10.1080/21691401.2018.1479709

**Published:** 2018-06-29

**Authors:** Eleonore Fröhlich

**Affiliations:** Center for Medical Research, Medical University of Graz, Graz, Austria

**Keywords:** Alternative *in vitro* models, co-culture, nanoparticles, inhalation exposure, oral exposure, intravenous exposure

## Abstract

Humans are exposed to a wide variety of nanoparticles (NPs) present in the environment, in consumer, health and medical products, and in food. Conventional cytotoxicity testing compared to animal testing is less expensive, faster and avoids ethical problems at the expense of a lower predictive value. New cellular models and exposure conditions have been developed to overcome the limitations of conventional cell culture and obtain more predictive data. The use of three-dimensional culture, co-culture and inclusion of mechanical stimulation can provide physiologically more relevant culture conditions. These systems are particularly relevant for oral, respiratory and intravenous exposure to NPs and it may be assumed that physiologically relevant application of the NPs can improve the predictive value of *in vitro* testing. Various groups have used advanced culture and exposure systems, but few direct comparisons between data from conventional cultures and from advanced systems exist. *In silico* models may present another option to predict human health risk by NPs without using animal studies. In the absence of validation, the question whether these alternative models provide more predictive data than conventional testing remains elusive.

## Introduction

Paracelsus, who was a physician, alchemist and astrologer, discovered that every substance can act as poison at a sufficiently high concentration and led to the concept of dose-dependent toxicity. Chemicals, environmental toxicants and medical products are subjected to toxicity testing, which is, in general, performed according to guidelines of regulatory agencies such as Organization for Economic Co-operation and Development (OECD), International Health Organization (ICH) & World Health Organization (WHO) and Food and Drug Administration (FDA). An important part of all studies is toxicity testing for the approval of drug compounds. Routine preclinical toxicity testing is time-consuming and expensive and still many drugs fail in early clinical phases not only due to lack of efficacy (43%) but also due to toxicity (33%; [1]). If the type of toxicity is further classified, hepatotoxicity (∼50%) is the most common, followed by cardiovascular and haematological problems (∼20% each) and by adverse immune effects (∼15%). The gold standard of toxicity testing is the assessment in animals, but since several years, the use of *in vitro* experiments instead of animal experimentation is encouraged. The idea of Reduction, Refinement and Replacement (3Rs) of animal experiments has first been published in 1959. In 2010, the European Commission requested the partial and even full replacement of animal studies. According to the US National Research Council, toxicity testing in the twenty-first century is carried out largely, but not entirely, without the use of animals. Although full replacement of animal studies appears not very realistic from the current perspective, various initiatives have been started to achieve this goal. Testing of tissue explants and tissue sections (*ex vivo* exposure) can reduce the use of animals. In addition, many strategies aim to improve *in vitro* exposures by developing physiologically more relevant culture conditions using co-culture of various cell types, culture in three-dimensional (3D) systems, and application of flow and mechanical stimulation. Specific questions can be addressed by testing of isolated organelles.

## Alternative toxicity testing methods

*Ex vivo* and *in vitro* studies are options to replace animal exposures and their use varies depending on the exposure route or tissue under investigation. The extent of use of *ex vivo* samples is linked to the epithelial barrier to be assessed. Protective epithelia are thick and relatively robust because they have to protect the body from mechanical and chemical damage and invasion of pathogens. Receptive barriers, by contrast, serve to absorb nutrients and exchange gases. To fulfil these functions, they are thinner, more permeable and more fragile. *Ex vivo* samples and commercially available reconstructed tissues are frequently used for skin permeability studies. The epidermis is a typical example for a protective barrier and excised skin samples maintain good barrier function for 24 h. Testing of irritation and corrosion with reconstructed epidermis is approved as an alternative to *in vivo* testing of cosmetics. The Cosmetics Directive of the European Commission provides the regulatory framework for the phasing out of animal testing for cosmetic purposes [2]. Reconstructed tissues of other protective barriers (oral epithelium and urogenital tract such as vagina) are commercially available (Supplementary Table S1), but few companies provide ready-to-use systems for organs such as liver, kidney and for receptive barriers (respiratory epithelium and intestinal epithelium). *Ex vivo* samples from these tissues typically remain viable only for short time. Viability of excised small intestine samples, for instance, declines already after 5 min [3]. Standardized toxicity testing has specific requirements: models should react very reproducibly to obtain high-quality data. In addition, it should provide the possibility to assess a higher number of samples in parallel, a process usually referred to as high-throughput screening (HTS). The model should also possess high predictive value to be able to replace or reduce *in vivo* experiments.

The isolated perfused liver has the highest predictive value for drug-induced liver disease, but tissues are different to obtain, viable only for a limited time span and not suitable for HTS. In general, there is an inverse relation between predictive value for toxicity in humans and ease of use, costs and potential for HTS analysis.

Compared to conventional compounds, the need for representative systems in the testing of nanoparticle (NP) toxicity is even higher because deposition on cells, permeation of acellular barriers, cellular uptake and change by the exposure conditions are more complex for NPs than for conventional compounds. Important issues in particle testing *in vivo* are listed in Table 1.

**Table 1.** Specific issues in the toxicity testing of NPs.

Parameter	Specific issues with NPs
Exposure medium	Exposure medium is important because particle parameters are changed by medium composition (agglomeration)
Duration of exposure	Usually too short as NPs are metabolized to lower extent than conventional compounds
Monolayer culture	NPs cross cell layers by diffusion and paracellular transport to lower extent than conventional compounds
Monoculture	Cell uptake differs between phagocytes and non-phagocytes for NPs and less for conventional compounds
Absence of dynamics	NPs get in contact with cells by sedimentation, which does not play a role for conventional compounds
Low cell differentiation	Secretion of mucus hinders permeation of NPs to higher degree than conventional compounds due to the size exclusion effect^a^

^a^Size exclusion means that NPs, due to their size, are sieved though the mucus mesh.

## Toxicity testing of NPs

**Figure 1.**
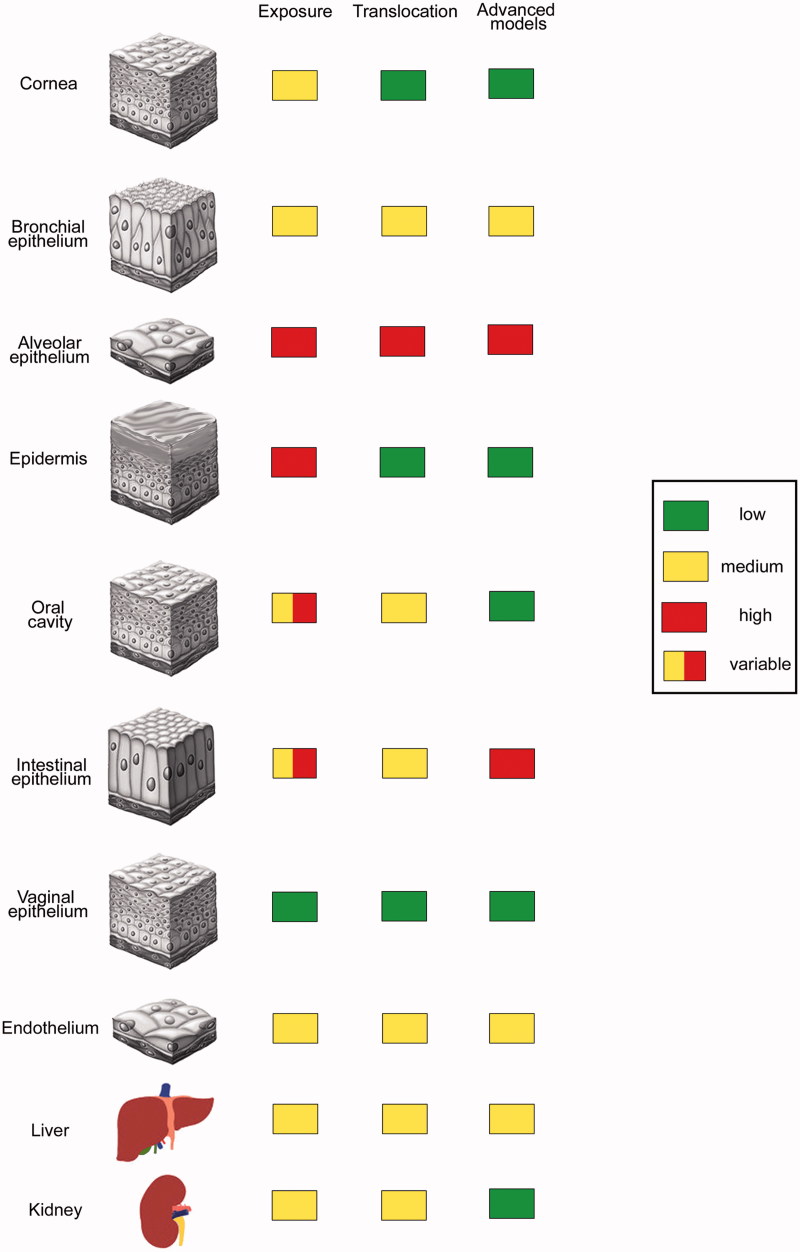
Extent of NP exposure, translocation and use of advanced cell culture models in the testing for epithelial barriers and internal organs. Independent from the extent of exposure use of *in vitro* models for protective barriers (cornea, epidermis, oral cavity, vaginal epithelium) is low as good *ex vivo* systems are available. *In vitro* systems are used when particle exposure is high and robust *ex vivo* systems are missing (alveolar and intestinal epithelium).

Humans are exposed to NPs by the environment (air, soil and water), consumer products and food, products of daily life and medicine. Not only the extent of exposure but also translocation and relevance of *in vitro* models differ between the portals of entry (Figure 1). As absolute doses differ between particles and exact exposure concentrations are mostly unknown, doses are classified as low, intermediate and high in Figure 1. Toxicity is further determined by the permeability of the respective barrier, which is indicated in the same way. Numerous animal and *in vitro* studies demonstrated adverse biological effects of NPs, but the predictive value of these data for the human situation is still unclear. Part of the problem is due to lack of knowledge about realistic exposure levels. The use of unrealistically high exposure doses in the experiments as well as anatomical and physiological differences between animals and humans limits the value of data acquired in animals. Toxicants applied by the most common application routes (skin, gastrointestinal (GI) tract, lung, blood) cause different effects and testing of all NPs in animals appears unrealistic due to time, ethical and financial concerns. *In vitro* testing is faster, less expensive and poses no ethical problems. Routine toxicity screening, however, even when using human cells, does not mimic the situation of cells *in vivo* because immortalized cells in monoculture are cultured on plastic surfaces at high concentrations of oxygen and glucose. Therefore, conventional culture induces changes in the cell-specific phenotype due to the absence of important physiological stimuli, such as the presence of a basement membrane and supply with nutrients from the basal side, absence of mechanical stimuli, static condition, and lack of interaction with other cells. Many immortalized cells in such culture possess only a part of the functional capacity that the cells, they are derived from, expressed *in vivo*.

## Status of *in vitro* testing of target tissues for particle toxicity

Relevant barriers/organs for NP exposure include epidermis for dermal exposure, oral cavity, small and large intestine for oral uptake, bronchial and alveolar epithelium for inhalation, and endothelium for intravenous exposure. Particle accumulation was seen mainly in liver, lung and kidney, but histopathological changes were also reported for bone marrow and spleen [4]. *In vitro* assays are used to a different extent to reveal damage to these tissues.

Similar to cosmetics, dermal exposure to NPs can be assessed by *ex vivo* samples and commercially available reconstructed skin. *Ex vivo* samples of intestinal epithelium, alveolar epithelium and endothelium have shorter survival times and are better studied *in vitro*. *Ex vivo* samples of liver and kidney have only a limited survival time and the use of *in vitro* models is quite common. Bone marrow toxicity, haematotoxicity or myelotoxicity can be predicted by the colony forming unit (CFU) assay using either murine or human primary bone marrow cells. The assay is technically challenging because specificity and sensitivity are determined by various factors, mainly cell number and growth factor cocktail. Once established, the predictive value for myelosuppression is high for conventional compounds. Mainly granulocyte–macrophage lineage is assessed and this assay in modified form can also be used for NPs. However, only few data from NPs are available so far. A study on several NPs in the size range of 20–200 nm showed that antimony oxide (Sb_2_O_3_) and cobalt (Co) affected human granulocyte–monocyte lineage and erythroid lineage [5]. Silver (Ag), gold (Au), iron oxides, (Fe_2_O_3_ and Fe_3_O_4_) and titanium dioxide (TiO_2_) caused no adverse effects. Thrombocytotoxicity can also be assessed using the CFU assay, but data on NP effects are missing so far. Pathological changes in spleen histology may indicate effects on the immune system. Possibilities to assess immune effects *in vitro* are limited. Further information on the value of *in vitro* testing of NPs can be found elsewhere (e.g. [6]).

Physiologically relevant *in vitro* models have to fulfil several requirements, which include, on the one hand, appropriate culture and cell composition (e.g. cellular phenotype and co-culture) and, on the other hand, specific exposure conditions (e.g. suspension in physiological fluids and application as aerosol).

This review focuses on the role of *in vitro* models in toxicity testing of NPs, without addressing the role of *ex vivo* systems and organelle testing.

## Cell differentiation and cell diversity in culture

Conventional culture lacks intense cell–cell interaction, signalling molecules and mechanical effects/dynamics. Furthermore, routine cytotoxicity testing is performed on subconfluent cells, a situation different from *in vivo*, where epithelial cells (intestinal, endothelial, respiratory, parenchymal cells of liver and kidney, etc.) are in direct intercellular contact and, with the exception of cells of the intestine, not constantly proliferating. Although cell lines show a decreased state of differentiation, they are preferred for basal toxicity screening because they possess all basal cellular functions and react in a more reproducible way than primary cells. To produce reliable data, cell lines have to be well characterized and to be routinely screened for bacterial contamination and for cross-contamination. Origin and use of cell lines in the different models mentioned in this review are provided in Table 2. To address cell-specific toxicity, cells need to express the specific phenotype and need to be treated in a specific way.

**Table 2.** Origin and use of cell lines in the physiologically relevant models.

Cell line	Species	Origin	Use
16HBE14o–	Human	SV40 immortalized bronchial epithelial cells	Bronchial epithelium, toxicity
A549	Human	Lung carcinoma	Alveolar epithelium, toxicity
BEAS-2	Human	Epithelial virus transformed bronchial epithelial cells	Bronchial epithelium, toxicity
Caco-2	Human	Colorectal adenocarcinoma	Intestinal epithelium, barrier function, toxicity
CAL27	Human	Oral squamous cell carcinoma	Cancer cell
Calu-3	Human	Lung adenocarcinoma	Bronchial epithelium, barrier function
CRL-2102 (C2BBe1)	Human	Clone of Caco-2 cells	Enterocytes
EAhy926	Human	Fusion of HUVEC with human pulmonary adenocarcinoma A549 cells	Endothelium
Fa2N4	Human	SV 40 immortalized hepatocytes	Hepatocytes
hAELVI	Human	Lentivirus immortalized alveolar epithelial cells	Alveolar epithelium, barrier function
HeLa	Human	Cervical cancer	Cancer cell
Hep3B	Human	Hepatocellular carcinoma	Hepatocytes
HepaRG	Human	Liver progenitor cells	Hepatocytes
HepG2 Hep2/C3a	Human	Hepatocellular carcinoma derived from HepG2 cells	Hepatocytes
HK-2	Human	Proximal tubule papilloma	Renal tubule cells, barrier function
HMC-1	Human	Mast cell leukaemia	Mast cells
HPMEC-ST1.6R	Human	Virus transfected microvascular endothelial cells	Endothelial cells
HT29 and HT29-MTX	Human	Colon adenocarcinoma cells and cells treated with methotrexate to induce mucus production	Goblet cells
Huh7	Human	Hepatocellular carcinoma	Hepatocytes
ISO-HAS 1	Human	Haemangiosarcoma	Endothelium
J774.A1	Mouse	Reticulum cell sarcoma	Monocytes/macrophages function
LLC-PK1	Pig	Kidney cells	Renal tubule cells, barrier function
LS174	Human	Colorectal adenocarcinoma	Intestinal epithelium
LS513	Human	Colorectal carcinoma	Intestinal epithelium
M5076	Mouse	Ovarian sarcoma	Cancer cells
MCF-7	Human	Breast adenocarcinoma	Metabolization, action of transporters
MDCK	Dog	Distal renal tubules	Renal tubule cells, barrier function
MG63	Human	Osteosarcoma	Osteoblasts
MH-S	Murine	Simian virus 40 transformed alveolar macrophages	Alveolar macrophages
MLE 12	Mouse	Lung epithelial cells	Alveolar epithelium
MRC-5	Human	Foetal lung fibroblasts	Fibroblasts
NCI-H322	Human	Bronchoalveolar carcinoma	Alveolar epithelium, toxicity
NCI-H441	Human	Papillary lung adenocarcinoma	Alveolar epithelium
NCI-H460	Human	Large-cell lung cancer	Cancer cell
NIH/3T3	Mouse	Embryonal fibroblasts	Fibroblasts
NKi-2	Human	hTERT immortalized proximal tubule cells	Proximal renal tubule cells
NRK52K	Rat	Kidney epithelial cells	Renal tubule cells, barrier function
Raji B	Human	Burkitt’s Lymphoma	Induction of M cell formation in co-culture
Rat-2	Rat	Foetal fibroblasts	Fibroblasts
RAW 264.7	Mouse	Abelson murine leukaemia virus-induced tumour	Monocytes/macrophages function
T84	Human	Colorectal carcinoma	Intestinal epithelium
THP-1	Human	Acute monocytic leukaemia	Monocytes/macrophages function
TK6	Human	Hereditary spherocytosis lymphoblasts	Genotoxicity testing
TLT	Human	Macrophages	Macrophages
U937	Human	Histiocytic lymphoma	Monocytes/macrophages function

Liver models should express metabolizing enzymes representative for hepatocytes and kidney models the typical transporters of the proximal tubule epithelial cells, which are mainly responsible for drug excretion. For endothelial cells, the presence of cell-specific adhesion molecules and uptake routes for a realistic estimation of particle uptake are required. To provide the required characteristics of the model, several strategies have been tried. Many cells increase differentiation when grown in an apolar environment, either on membranes, on scaffolds or as scaffold-free spheroids. Culture at an air–liquid interface (ALI) is the most representative method for respiratory cells. In this culture, cells are supplied with medium only from the basolateral side, whereas the apical side is facing air. To induce endothelial differentiation, flow systems providing appropriate shear stress are used. Various cell types (e.g. hepatocytes and osteoblasts) respond to mechanical stimulation induced by sandwich culture, cell sheet engineering or dorsal stimulation (e.g. by atomic force microscopy) with increased cell differentiation.

**Figure 2.**
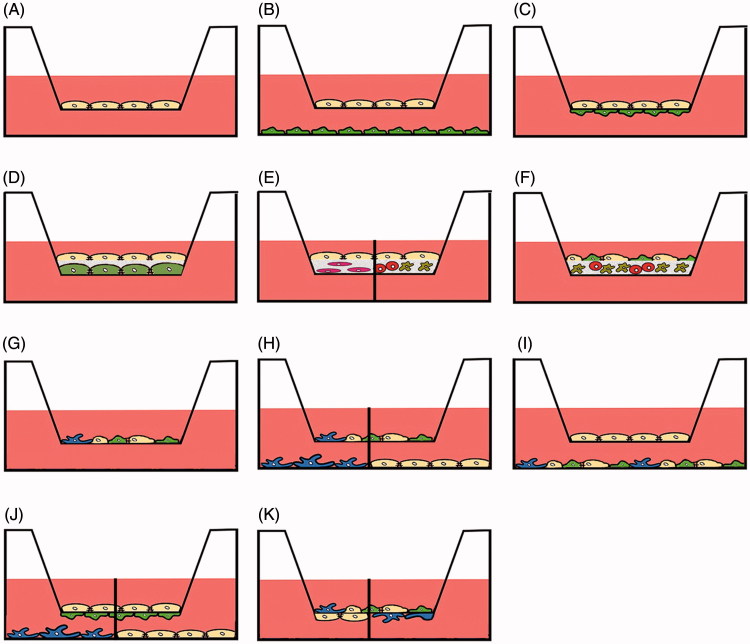
Use of transwell membranes in advanced culture models. Monoculture for permeation experiments (A), indirect contact (B) and direct or indirect contact (C) co-culture of only one cell type in each chamber. Cells can be cultured or separated by matrices that may either be acellular (D) or contain one (E) or several types of cells (E, F). Co-culture systems may consist of two and more cell types in the apical compartment (G), co-culture of two and more cell types in the apical compartment in indirect culture with one cell type in the basolateral compartment (H), co-culture of one cell type in the apical and several types of cells in the basolateral compartment (I), combined direct contact and indirect contact culture (J), direct contact culture of several cell types in the apical compartment and one type in the basolateral compartment (K). The separation line in H, J and K indicates that different cell types in monoculture can be used in the basolateral compartment or in the apical compartment (E).

Tissue-specific toxicity presents an additional challenge because interactions between cells have to be included. Intestinal models should be composed of goblet cells, immune cells and epithelial cells. Alveolar models should include alveolar epithelial cells and macrophages. Membrane-based systems are widely used in co-culture models of intestinal and respiratory barrier. Co-culture between two cells can be performed in the way that cells are separated by a membrane and can only exchange soluble factors (Figure 2(B)). There is also an option that a matrix layer (e.g. hydrogel, collagen, matrigel, etc.) separates different cell types or that a matrix layer containing cells is covered by epithelial cells (Figure (2D–F)). Models in which cells are cultured on opposite sides of a membrane (Figure 2(C)) may have direct contact or indirect contact because, depending on the pore size of the membrane, cells may interact across the membrane via processes. Fibroblasts grown on one side of a membrane with 1.2 μm pore size were capable of reaching and contacting other cells grown on the opposite side of the same membrane [7]. Smaller pore sizes usually allow only the exchange of macromolecules. Direct co-culture of cells can be used in the apical (Figure 2(D–H, K)) and basolateral compartments (Figure 2(I)).

Despite the many advantages and broad use, it has to be mentioned that the most often used membranes in transwell systems hinder the free passage of NPs. The effect depends on the membrane material and varies between particles. Particle permeation was more impaired for 0.4 µm than for 3 µm pore size and higher for polyester compared to polycarbonate membranes. The role of particle surface charge in hindrance to cross transwell membranes is not clear. Using the same membranes, Geys et al. [8] observed around 50% permeation of 50 nm carboxyl and amine-functionalized polystyrene particles, whereas Dekali et al. [9] reported retention of amine-functionalized and non-functionalized, 50 and 100 nm, polystyrene particles.

Different proliferation rates of the co-cultured cells may present a problem and limit the use of co-culture systems over longer time periods. In Caco-2/methotrexate-treated HT-29 (HT29-MTX) cell co-cultures, HT29 cells proliferate faster than Caco-2 cells, which results in the problem that the 9:1 ratio (Caco-2:HT29-MTX cells) rapidly changes over time. Instead of co-culturing cells in transwell systems, conditioned medium from one cell type can be added to another cell type at specific intervals. More recently, techniques were developed that enable a continuous exchange of media between cells. These techniques use small volumes and are summarized as microfluidics. Using continuous perfusion and chemical gradients, they represent better than conventional systems the microenvironment of cells *in vivo*. These systems are not yet established models for toxicity testing. A detailed description of the various microfluidics platforms is beyond the scope of this review and the reader is referred to reviews dedicated to this topic.

## Intestinal models

Oral exposure of humans occurs by NPs in food, health and medical products (Figure 1). The exposure may show pronounced inter-individual variability as diets vary and specific food contains particularly high levels of NPs [10]. Stomach models are not widely used in pharmaceutical and toxicological testing because, compared to the intestine, little absorption takes place in stomach. Testing for intestinal permeability, on the other hand, is a routine in the evaluation of oral drugs. *Ex vivo* samples and small intestine samples, mostly from rats and mice, are employed for the assessment of permeability of conventional compounds while porcine samples are more rarely used. As tissue viability decreases fast, cell culture models are required when active uptake mechanisms and effects of >2 h are studied.

Caco-2 cells are the most often used cellular model for the assessment of drug absorption across the small intestine. Permeability determined in Caco-2 monolayers (Figure 2(A)) correlates well with *in vivo* absorption of the respective drugs and the model is accepted by the European Centre for the Validation of Alternative methods as replacement for *in vivo* permeability. Instead of Caco-2 monolayers on membranes an openable artificial microtube device coated with Caco-2 cells to evaluate absorption oc compounds has been suggested [11]. Caco-2 cells possess microvilli, express several enzymes of oxidative drug metabolization by cytochrome P450 (CYP) isoenzymes as well as a variety of uptake and efflux transporters (MRP2–6, BCRP, OATP1A2, OATP2B1, OCT1 and PEPT1). They differ from enterocytes of the small intestine by lack of mucus production and lack of CYP3A4 expression, the CYP P450 isoenzyme most relevant for drug metabolization. In contrast, they form tighter intercellular junctions, characterized by higher transepithelial electrical resistance (TEER) values, than epithelial cells of the small intestine. This leads to a lower permeability of hydrophilic molecules. In general, TEER values correlate with permeability of small molecules, but peristalsis in combination with flow increased drug permeability across Caco-2 monolayers leaving TEER values unchanged [12]. By providing additional stimuli, Caco-2 cells can differentiate into complex structures and give rise to different cell types. When cultured in hydrogels, Caco-2 cells form villi and integration of mechanical stimuli causes differentiation into goblet cells, enteroendocrine cells and Paneth cells as the main cell types of epithelium of the small intestine [13]. Caco-2 cells and several other cell lines (LS174, LS513 and HT29 cells) produce confluent monolayer with mucus production when they are cultured in semi-wet condition in combination with mechanical stimulation [14]. T84 cells originate from colon carcinoma tissue, form a tight epithelial barrier, produce mucus and are rather used as model for the large intestine. Their CYP450 enzyme expression, however, is lower than that of HT29 and Caco-2 cells; therefore, the combination of Caco-2 cells with HT29-MTX cells is preferred as model for oral bioavailability, where metabolization at the intestinal barrier is also involved [15].

Since NPs cross the intestinal barrier of the small intestine mainly via Microfold (M) cells, Caco-2 monocultures may underestimate permeation. M cells perform transcytosis of antigens across the gut epithelium and play a major role in the induction of efficient immune responses. *In vitro*, M cells are generated by co-culture with Raji B cells and Caco-2 cells [16]. By combination with mucus-producing HT-29-MTX cells, Caco-2 cells and Raji B cells in direct co-culture form a physiologically relevant model [17–19], Figure 2(G). These models are exclusively based on the use of human cells, but rodent cells/human cell combinations also exist. Rat 2 fibroblast-like cells embedded in matrigel-supplemented collagen gel and overlaid with CRL-2102 human colon carcinoma cells were used by Viney et al. [20], Figure 2(E). Another co-culture model uses Caco-2/HT29-MTX cells on collagen gel containing THP-1 cells and mouse embryonic fibroblasts [21], Figure 2(E). As no immunological parameters were assessed in these studies, potential activation of immune cells by cells from a different species cannot be excluded. Models for the evaluation of immune effects, on the other hand, consist exclusively of human cells. One model combines H4–1 small intestinal cells in the apical compartment of a transwell with TLT human monocyte/macrophages in the basolateral compartment [22], or Caco-2 cells in the apical compartment and peripheral blood mononuclear cells (PBMCs) in the basolateral compartment [23], Figure 2(C). Other models for immune responses comprise Caco-2 cells and dendritic cells (DCs) and/or macrophages, derived from, for example, periphery blood monocytes [24,25]. Co-culture of Caco-2 in the apical and U937 macrophages in the basolateral compartment has been used in microfluidic chambers [26], Figure 2(C). A model consisting of Caco-2 cells cultured on top of a collagen gel containing PBMC-derived DCs and macrophages was designed to study specifically inflammatory processes in the intestine [27], Figure 2(E). Colon mucosa can be constructed by culturing Caco-2 cells and HT29-MTX cells on collagen gels containing primary fibroblasts and differentiated THP-1 cells [21], Figure 2(E). Caco-2 cells with and without mucus overlay with porcine mucin and Caco-2/HT29-MTX co-cultures (Figure 2(A,G)) were compared regarding permeability of iron [28]. In this study and in another study by Vazquez et al., mucus decreased the permeability of metal ions [29], indicating that mucus presents an independent barrier for oral absorption. Barrier function of mucus for NPs has been demonstrated *in vitro* and *in vivo*.

To address metabolization, co-culture of Caco-2 cells, HepG2/C3a liver cells and human breast carcinoma MCF-7 cells were performed by culturing cells in separate compartments, connected through microfluidic channels. Esch et al. [30] coupled a fluidic GI (Caco-2/HT29-MTX) and liver (HepG2/C3a) tissue microphysiological system to investigate the effects of ingested NPs on the liver.

## Models for the alveolar barrier

Exposure of humans to particles is high because inhalation of air-borne substances (dust, pathogens, chemicals, etc.) is unavoidable (Figure 1). In addition, there is also exposure to inhaled drugs as respiratory diseases have a high prevalence. Although the primary sizes of therapeutically inhaled particles are not in the nanometre range (1–5 µm), smaller particles are generated by dissolution. Particles of 20 nm deposit to 50% in the alveolus, the most permeable region of the respiratory epithelium, and to 25% in the head and tracheobronchial regions [31]. Toxicity testing in *ex vivo* models (“perfused lung”) is not common because models are technically demanding and remain viable only for short time.

Calu-3, BEAS-2B and 16HBE14o cells are the most commonly used bronchial epithelial cell lines for *in vitro* testing [32]. In addition, commercially available reconstructed bronchial epithelium (EpiAirway™ MatTek Cooperation, Ashland) can be used. MRC-5 fibroblasts embedded in a collagen matrix on transwell membrane, covered with PBMC-derived DCs and 16 HBE16o bronchial epithelial cells as the top layer, have been used for testing of NPs [33], Figure 2(F). Calu-3 cells and THP-1 cells in the apical compartment were cultured together with endothelial cells in the basolateral compartment [9], Figure 2(K).

A549 cells are the most frequently used cells for the assessment of alveolar toxicity ( [34]). This is due to the fact that they express the same pattern of metabolizing phase I (cytochrome P450 isoenzymes) and phase II enzymes (transferases) as lung tissue. Enzyme activities can be increased by the typical inducers of CYP450 enzymes, such as dexamethasone and phenobarbital. The enzyme expression profile of NCI H322 cells is less similar to human lung tissue although they are derived from the main metabolizing cells of the human lung, the Club or Clara cells. The main disadvantage of A549 cells is the absence of a good barrier function. Another epithelial cell line, the H441 alveolar cells, is also being used. Upon stimulation with glucocorticoids, the cells reach higher TEER values than A549 cells but still do not possess the barrier function of alveolar cells *in vivo*. Only recently, a cell line has been developed which presents alveolar barrier function and can also be cultured in ALI culture [35]. Human alveolar epithelial lentivirus immortalized (hAELVi) cells morphologically resemble alveolar type I cells, produce surfactant and express high levels of metabolizing enzymes and transporters.

The presence of alveolar macrophages is very relevant for NPs because phagocytes can ingest NPs to higher extent than non-phagocytic cells [36]. Furthermore, lung macrophages are important regulators of inflammatory processes in the lung. Several co-culture systems addressing NP effects at the alveolar barrier have been published. A549 cells were cultured together with human monocyte-derived macrophages in the apical compartment of a transwell chamber and human monocyte-derived DCs on the other side of the membrane in the basolateral compartment [37,38], Figure 2(K) or a mixture of A549 alveolar epithelial cells + THP-1 monocytes + HMC-1 mast cells (ratio 10:2:1) in the basolateral compartment and an insert containing EAhy926 endothelial cells in the apical compartment [39], Figure 2(I), was used. The model by Klein et al. also consisted of A549 cells, HMC-1 mast cells, THP-1 monocytes and EAhy926 endothelial cells and differs from the previous one in the way that endothelial cells were seeded on the basal side of the transwell, and A549 + THP-1 and HMC-1 cells seeded on the apical side of the membrane [40], Figure 2(K). The commercially available reconstructed alveolar epithelium EpiAlveolar™ is composed of alveolar epithelial cells + endothelial cells.

Microfluidics systems used A549 cells cultured on suspended polyethylene terephthalate (PET) membranes to create ALI conditions and characterized the physiological potential of the cells [41]. After 3 weeks, the cells showed indication for reduced surface tension compared to submersed cells. Tension decreased from 42 to 37 mN/m in submersed culture and from 39 to 29 mN/m in ALI culture. TEER values of the microfluidic system were similar to conventional transwell cultures in the respective condition and reached 177–195 Ω cm^2^ in ALI and 147–152 Ω cm^2^ in submersed culture.

## Endothelial models

Endothelial cells get in contact with NPs in medical products and, to a minor extent, by translocation of inhaled or ingested NPs (Figure 1). Endothelial cells restrict access of NPs to internal organs and regulate inflammation and coagulation in blood. Their phenotype is markedly influenced by the culture conditions and shear stress is the main stimulator of surface marker expression and morphology. Effects on large vessels (arteries and veins) can be studied using endothelial monolayers on transwell membranes with endothelial cells and smooth muscle cells (SMCs) cultured either on one or on opposite sides of the membrane (Figure 2(C,G)). SMCs can also be cultured on the bottom of the transwell and endothelial cells on top of the membrane (Figure 2(B)) and lastly, endothelial cells can be cultured on top of a collagen gel containing SMCs [42], Figure 2(E).

In contrast to intestinal and respiratory barrier, endothelial models are frequently primary cells, mainly human umbilical vein endothelial cells (HUVEC). These cells can relatively easily be obtained, and pooling cells isolated from several umbilical cords can reduce differences between donors. Also the use of primary endothelial bovine aortic endothelial cells and primary porcine artery endothelial cells is quite common. Among the best-characterized human endothelial cell lines are EAhy926 cells as representatives for macrovascular endothelium and HMEC-1 cells for microvascular endothelium [43]. EAhy926 cells have also been included in co-culture models of the respiratory barrier [39,40].

The blood–brain barrier is one of the most studied and tightest barriers of the human body and many different *in vitro* models have been developed oxicity testing with these models is not common. NPs do not easily enter the brain and strategies to increase crossing of the blood–brain barrier for medical treatment do not result in high permeation rates. Only at extremely high concentrations, effects in the brain were observed. Ag, Al and Cu NPs injected at a concentration of 30 mg/kg in rats destroyed the blood–brain barrier [44]. These doses correspond to 0.7 mg (Ag, Al or Cu NPs)/ml plasma in humans (according to body weight and plasma volume of the standard man, http://www.physiologyweb.com/figures/physiology_illustration_tPksfgTyDcZ10zEq1Wp1FqLjrBRL8IGL_body_fluid_compartments_of_a_70_kg_adult_man.html) and are not realistic for human exposure.

## Liver models

NPs reach the liver by intravenous exposure and by uptake through the GI tract (Figure 1). Models have to express liver-specific functions, which comprise synthesis of glucose, serum proteins and urea, and metabolization of endogenous and exogenous substrate by oxidation, reduction, hydrolysis, hydration, conjugation, condensation or isomerization. In the context of toxicity testing, dehydrogenases of the CYP P450 isoenzyme family are a key parameter in the evaluation of the liver model because of the great importance in metabolization of endogenous and exogenous substrates. Conventional culture of primary hepatocytes leads to loss of cell polarity and of specific hepatocyte function but coating of the growth substrate, co-culture with endothelial cells, fibroblasts or different non-parenchymal liver cells can improve hepatocyte function in primary hepatocytes and hepatocyte cell lines [45]. Natural hydrogels consisting of either chitosan, alginate, collagen or Matrigel® and synthetic hydrogels based on polyvinyl alcohol (PVA), polyethylene glycol (PEG) in combination with poly(lactic-co-glycolic) acid (PLA), heparin or Arg-Gly-Asp peptide, or PuraMatrix™ increased CYP enzyme activities and capacity to secrete albumin and urea. The scaffolds preserved CYP enzyme activities for longer time span than conventional culture systems. A variety of bioreactors, hollow fibre-based, alginate encapsulates, multichamber modular systems (Quasi-Vivo®, Liverchip, Hepachip, 3^D^-KITChip) combine 3D environment and perfusion. These models are currently not used in toxicity testing due to low donor availability and high inter-individual variations. Transwell-based systems are also used for *in vitro* liver models. Primary hepatocytes on membranes of transwells or in plastic wells overlaid with matrigel and endothelial cells as the top layer can serve as liver models [46], Figure 2(D). Alternatively, endothelial cells on matrigel-coated filters and hepatocytes on the other side of the membrane form also functional liver models (Figure 2(C)). Considering the higher uptake of NPs by hepatic stellate cells (Ito cells) than by hepatocytes co-cultures of both cell types is important. Hepatocytes in the basolateral compartment and co-culture of stellate and macrophages in the apical compartment either in indirect or in direct contact [47] (Figure 2(H,J)) have been used. In other models, the inclusion of rat hepatocytes and stellate cells in spheroids increased CYP450 expression [48]. Microfluidic platforms of hepatocytes and stellate cells have mainly been used in studies on hepatic fibrosis.

Although not an optimal hepatocyte model, HepG2 cells are most often used in conventional hepatotoxicity testing. The cells have the capability to secrete liver-specific plasma proteins but expression of metabolizing enzymes is low [49]. Other hepatocyte cell lines, such as Hep3B, Huh7 and Fa2N4 cells, have even lower metabolic capacity. HepaRG cells, derived from a hepatocarcinoma, represent a mixture of terminally differentiated hepatocyte- and cholangiocyte-like cells [50]. The cells show good expression of CYP450 isoenzymes in conventional culture and form bile canaliculi-like structures when seeded together with primary hepatic stellate cells [51].

Flow condition, 3D environment and conditioned medium from other cells appear to be of critical importance for generation of functional liver models. There are, however, also data that cast doubt on the importance of these parameters. A comparison of different long-term 3D and two-dimensional (2D) culture systems showed that CYP isoenzyme activities increased over time independent of the culture condition [52]. The authors postulated that the increase in metabolic competence of HepG2 was more due to prolonged culturing than to different stimuli in 2D and 3D condition.

NPs inhibited the activity of CYP P450 isoenzymes in microsomal preparation and conventional cell culture studies, but it is questionable that the required concentrations are achieved *in vivo* [53–56]. These results need confirmation in more realistic exposure scenarios.

## Renal models

NPs reach the kidney after intravenous exposure (Figure 1) and may damage tubular epithelium and glomeruli [57]. Isolated perfused kidney, precision cut renal slices, isolated tubules, primary cells and cell lines can be used for evaluation of excretion and renal toxicity. Advantages and limitations of these models are similar to liver. The isolation of the functional units, glomeruli or renal tubules, is difficult and the subsequent culture is highly sophisticated. Common screening for kidney-related toxicity addresses excretion and transporter function using monolayers of primary renal proximal epithelial cells or cell lines from different species (e.g. HK-2, NKi-2, LLC-PK1, MDCK, NRK-52K cells) cultured on transwell membranes. Renal toxicants, such as cisplatin, can be identified using this technology although no cell line displays all features of renal proximal tubular epithelial cells [58]. Microfluidics systems are also used for renal toxicity testing. Kidney on a chip toxicity testing focuses on assessment of proximal tubule function. In one of the rare studies on NP effects, isolated proximal renal tubules were used to assess uptake and transport of quantum dots [59].

## Relevance of advanced cell culture models for NP testing

In the following sections, differences between advanced and conventional culture conditions, which might lead to different responses to NPs, will be discussed. General differences in oral and respiratory exposure include the fact that, due to the missing of acellular layers (surfactant, mucus), NPs may reach intestinal and respiratory cells in higher concentrations. The high proliferation rate compared to 3D culture may decrease intracellular levels of NPs in conventional culture. Monocultures lack the influence of cytokines secreted by cells in co-culture. Data obtained in advanced cultures (3D culture, co-culture, mechanically stimulated culture) will be compared to conventional culture.

### Effects of 3D environment

The culture in a 3D environment, usually on membranes, microcarriers, scaffolds or in hydrogels, affects cell proliferation. The potential mechanism is the greater cell-to-cell contact area compared to 2D culture which usually induces growth/contact inhibition. The lower anti-tumour activity of most chemotactic drugs in 3D than in 2D culture most likely is due to a reduced proliferation rate of cells in 3D culture because cytostatic drugs act more potent on proliferating cells [60]. The link to proliferation is more obvious when cells with higher proliferation in 3D culture are included. Oral cancer CAL27 cells showed a higher proliferation rate in spheroid than in conventional 2D culture and were also more sensitive to docetaxel, bleomycin and erlotinib in 3D [61]. Inhibition of proliferation by 3D culture may also explain why HepG2 cells expressed similar levels of CYP isoenzymes when cultured in 3D (embedding in Matrigel, Alvetex or collagen) and 2D culture after the same culturing time [52]. Various studies reported higher cytotoxicity of NPs in 2D than in 3D culture. Pluronic F68 and BSA-coated single-walled carbon nanotubes (SWCNTs) acted toxic only in 2D cultures of THP-1 cells [62]. Toxicity of carbon nanotubes was much higher in 2D culture of EAhy926 cells than in 3D microcarrier cultures [63]. HeLa cells and osteosarcoma MG63 cells reacted more sensitive to bismuth (Bi), Bi-NH_2_, Bi-PEG and Bi@SiO_2_ NPs in 2D than in spheroid culture [64]. Finally, CdTe NPs acted much less toxic on HepG2 cells cultured in spheroids than in 2D cultures. In addition to the extent, the type of cell death induced by the exposure was different [65]. Apoptosis was more pronounced in spheroid culture, particularly in the centre of the spheroid. In 2D culture, necrosis was the predominant type of cell death. Access of nutrients, toxicants and particles to viable cells is presumably lower in 3D than in 2D culture. The reduced access may particularly affect NPs, which typically cross cell layers only to a small extent. The decreased particle concentration in the centre of the spheroid was the reason for the change in cell death. The theory about restricted access of toxicants to cells is further confirmed by the findings that cytotoxicity of ZnO NPs on A549 cells grown in spheroids as loose aggregates was higher than in 2D culture. NIH3T3 fibroblasts formed dense aggregates and showed a similar reaction to exposure with ZnO in 3D and in conventional culture [66]. The reduced access of NPs to viable keratinocytes in the basal layer of the reconstructed epidermis may contribute to the lower genotoxicity of 16 and 86 nm silica particles in EpiDerm™ constructs compared to TK6 cells [67].

Concentration-dependent differences between 2D and 3D culture were identified for the action of ZnO (24, 56 and 87 nm) in Caco-2 cell cultures [68]. High concentrations of NPs induced more cytokine release, inhibition of proliferation, cell death and ROS generation in 2D than in cells embedded in agarose gels. At low concentrations, the opposite effect was seen and 3D cultures reacted more sensitive to ZnO NPs than 2D cultures. The 5 and 30 nm particles in pegylated and plain form caused higher toxicity at low concentration in 3D (alginate scaffolds) than in conventional 2D culture of primary porcine aortic endothelial cells [69]. Extrapolation of effects obtained in 2D to 3D culture is further complicated by the fact that the culture did not affect all particle effects to the same extent. Differences between ZnO-induced effects in 2D and 3D cultures were small for proliferation, time-dependent for cytokine release (12 h: 3D > 2D; 24 h: 2D > 3D) and prominent regarding type of cell death different in 2D and 3D (necrosis more in 2D and apoptosis in 3D).

As mentioned in section “Cell differentiation and cell diversity in culture”, membranes affect the passage of NPs and the use of scaffolds may introduce additional (artificial) effects. It has been reported that hydrogels restrict the diffusion of 130 nm iron oxide NPs [70]. Hindrance of particle diffusion through extracellular matrix and basal membranes is likely to occur also *in vivo*, but it is not clear whether scaffold and extracellular matrices restrict NP motion in a similar way. Cells in scaffold-free spheroids produce extracellular matrix themselves, which may be more similar to the situation *in vivo*. The advantage of the use of synthetic scaffolds is that they can be produced in different stiffness and can mimic the soft extracellular matrix of hepatocytes and the stiffer environment of osteoblasts. Perfusion can enhance or compensate the effect of scaffolds depending on size and functionalization of NPs. Penetration and uptake of 100 nm and 500 nm carboxyl polystyrene particles by cells embedded in hydrogel were similar in perfusion and in static conditions [71]. Particles of 100 nm did not penetrate the gel to sufficient extent to reach cells, but 40 nm particles permeated to a higher extent under perfusion than under static condition. The effect of perfusion on particle effects in monolayer (endothelial) culture is discussed in section “Intravenous exposure”.

### Effects of co-culture

Differences in particle uptake between phagocytes and non-phagocytes are relevant for evaluation of NP toxicity. Exposure of a model consisting of A549 + THP-1 + HMC-1 + EAhy926 cells showed that only the phagocytic THP-1 cells ingested 50 nm silica particles [40]. Due to cell interaction by cytokines and chemokines, the uptake in one cell type can affect the reaction of other cells in the same culture. Activation of phagocytes increased the cytotoxic action of doxorubicin-loaded poly(alkylcyanoacrylate) (PACA) NPs in co-cultures of M5076 murine ovarian sarcoma cells and J774.A1 macrophages [72]. In this model, the sarcoma cells were cultured in the upper compartment and the macrophages in basolateral compartment of a transwell (Figure 2(B)). In a similar set-up, macrophages in co-culture increased the efficacy of doxorubicin-loaded poly(isobutylcyanoacetate) (BIPCA) NPs on H460 human lung cancer cells [73]. The particles were ingested by MH-S murine alveolar macrophages and it was hypothesized that secretion of various inflammatory cytokines by the macrophages caused the cytotoxic action against H460 cells. This is possible because cytokines such as TNF-α, MCF-1 and IL-6 show relevant interspecies activity. Similar effects were also reported for environmental NPs. Co, Cu and ZnO NPs induced more apoptosis in co-cultures of RAW 264.7 macrophages and murine MLE-12 alveolar cells than in the respective monocultures, suggesting a potentiating effect of the NPs by the macrophages in a similar manner as for the doxorubicin-loaded NPs [74].

Co-culture may also decrease the reaction to NPs in monoculture. This was observed in co-cultures of epithelial cells. H441 and ISO-HAS-1 cells together (Figure 2(C)) were less sensitive to 30 nm silica NPs than either cell in monoculture. Cytotoxicity and induction of oxidative stress was abolished in the co-cultures but inflammation markers IL-8 and IL-6 increased more in co-culture than in monoculture [75]. A similar alveolar cell/endothelial cell model composed of H441 cells in the apical compartment and HPMEC-ST1.6R endothelial cells at the opposite side of a transwell membrane in the basolateral compartment was used to mimic respiratory exposure to NPs (Figure 2(C)). CuO, TiO_2_ and particulate matter (PM) added to the apical compartment were able to modulate endothelial cell activity by pro-inflammatory cytokines released from the H441 cells but cytotoxicity was decreased [76]. Recently, this model was upgraded by the culture of THP-1 monocytes in the basolateral compartment [77], Figure 2(J). Addition of ZnO NPs to the apical compartment induced expression of activation markers in the endothelial cells by release of pro-inflammatory cytokines, IL-6 and IL-8.

Taken together, immune effects appear to be more pronounced in 3D and co-culture models while cytotoxicity is mainly decreased in these cultures. The situation is different when one cell type can provide protection against NP damage. In co-culture of Caco-2/HT29-MTX cells, cytotoxicity was decreased. The presence of mucus in the co-culture decreased IL-8 release induced by exposure to 20 and 200 nm Ag NPs compared to Caco-2 monocultures [78].

### Mechanical effects

Cells *in vivo* are subjected to various mechanical effects, shear stress (endothelial cells), extracellular matrix stiffness (neural tissue), stretching (breathing, muscle cells), cyclic strain, compression and interstitial flow (connective tissue, bone, cartilage). When cellular reactions were compared in the presence and absence of mechanical effects, a variety of parameters were different. In general, differentiation was increased for endothelial cells, osteoblasts from precursor cells, kidney cells, intestinal cells, chondrocytes and neurons, etc. Culture of cells under mechanical stimulation also changed the responses to certain NPs. Mechanical effects, in the form of flow condition, are most important for endothelial cells. Mechanical stress applied to endothelial cells reduced the uptake of amine-functionalized silica particles by HUVEC cells compared to the non-stretched culture while uptake of plain and carboxylated NPs was not affected [79].

## Exposure conditions

In a good culture model, physiologically relevant cell culture should be combined with application of NPs in the appropriate way. This is important because particles agglomerate and some particles dissolve differently in water, buffer, cell culture medium and simulated body fluids. Although cell culture medium is used most often, various simulated fluids, such as GI fluids for oral exposure and simulated lung fluid are available. Exposure as aerosol for alveolar exposure and flow condition using plasma-protein-coated NPs for intravenous exposure would be physiologically more relevant than application as suspensions and in static condition, respectively.

### Intestine

Appropriate exposure conditions can be adopted from pharmaceutical testing of drugs. Exposure solutions for pharmaceutical testing of oral drugs have to be prepared according to guidelines provided by PharmEU and United States Pharmacopeia. The use of buffer systems with pH of 6.8 is the basic requirement for dissolution testing *in-vitro*. More biorelevant media such as simulated gastric fluid, fasted state simulated intestinal fluid (FaSSIF) and fed state simulated intestinal media (FeSSIF) contain, in addition to a buffer system, either pepsin or the natural emulsifiers, lecithin and taurocholate. As size, agglomeration and surface modifications by intestinal fluids determine the cellular action of NPs, several simulated GI fluids were used to describe changes in particle parameters during passage of the GI tract. These particle suspensions, however, were not applied to cells due to the low biocompatibility of most biorelevant gastric and intestinal fluids. These media have been used to determine drug release from nanoparticular drug formulations [80]. While FaSSIF medium can be used to assess drug permeability because it does not induce cell damage [81], FeSSIF media contains a higher concentration of detergents and causes damage to Caco-2 cells. The TIM-1 system simulates the influence of mechanical forces in addition to the chemical composition of the fluids of stomach, duodenum, jejunum and ileum. While isolated porcine intestinal tissue can be exposed to the undiluted content of the compartments, Caco-2 cells in monoculture and in co-culture with HT29-MTX cells are damaged [82].

Pre-incubation of NPs with the respective simulated digestive fluids can be performed and addition of the pre-treated particles diluted in cell culture medium to cells can be used to avoid adverse effects of biorelevant GI fluids on cells. Such pre-treatment increased the uptake of polystyrene NPs in a Caco-2/H29-MTX co-culture model compared to the untreated particles [83]. In addition to GI fluids, also cells can alter particle properties. To address these changes, sequential incubation of NPs with various cell types using microfluidics systems can be used. Polystyrene NPs were added to a multi-organ system, where they passed through the Caco-2/H29-MTX module (GIM) prior to reaching the liver, or to a liver-only control device. The GIM prevented 90% of NPs from crossing the epithelial barrier, and the remaining NPs reached the liver module, inducing the release of aspartate aminotransferase (AST, an injury marker). This injury was observable at lower concentrations than in liver-only models, indicating that contact with Caco-2/H29-MTX cells made the particles more toxic [30].

### Respiratory system

Inhaled NPs *in vivo* reach mucus or surfactant by deposition and do not sediment on submersed cells. This is achieved best by culture of respiratory cells in ALI and application of NPs as aerosol. The exposure is technically demanding as bacterial contamination should be avoided and optimal culture conditions for cells (e.g. humid atmosphere, incubation temperature, provision with nutrients) should be provided. Furthermore, particles should be deposited in an atraumatic way. The developed exposure systems use gravitational cloud settling, impactation and electrostatic deposition. Commercially available systems are CULTEX®, CULTEX® RFS, VITROCELL® and Vitrocell® Cloud system. Manual systems such as MicroSprayer® Aerosolizer and Dry Powder Insufflator™-DP4 (Penn Century Inc, Wyndmoor) have been developed for intratracheal exposure of rodents but have also been used for *in vitro* delivery of aerosols [84,85]. Deposition of NPs in the NAVETTA model is induced by application of an electrostatic field and the effect of gravity was excluded by positioning the cells in inverted position [86]. Particle-specific efficacy of deposition is a common problem of all systems. Deposition of polystyrene particles in the Vitrocell^®^ system (VITROCELL Systems GmbH, Waldkirch) based on cloud settling, for instance, was markedly lower than that of carbon nanotubes [85]. Often delivery rates are quite low because particles adhere to exposure chamber, tubes, etc. Manual devices have other limitations. MicroSprayer^®^ Aerosolizer leads to deposition of fluid on the cells and the Dry Powder Insufflator™-DP4 can cause mechanical cell damage [87]. Application of NPs suspended in a very small volume of cell culture medium or simulated lung fluid may be an option to mimic the exposure conditions at the alveolar barrier without material loss in the application system and cell damage [88].

ALI-based exposure systems have been used for the toxicological evaluation of copper (Cu) NPs, carbon NPs, zinc oxide (ZnO) NPs, gold NPs, polystyrene NPs, cerium oxide (CeO_2_) NPs and laser printer emission particles but only few comparisons to submersed exposure have been published. When the culture consisting of A549 epithelial cells together with human peripheral blood monocyte-derived DCs and macrophage cells was exposed to low concentrations of Ag NPs by the air–liquid interface cell exposure (ALICE) system, cells in ALI condition reacted similar to cells exposed to AgNO_3_ in submersed condition [89]. Also aerosolized bortezomib particles in ALI exposure and dissolved bortezomib in submersed condition activated the IL-8 promoter of A549 cells to similar extent [90]. ZnO NPs, on the other hand, induced higher levels of the anti-oxidative enzyme HO-1 in A549 cultured in ALI than in submersed culture [91]. Polystyrene particles, which do not dissolve, acted more cytotoxic on A549 cells in ALI than in submersed condition [85]. Based on these data, the relevance of physiologically relevant exposure systems is not clear. It is possible that the extent of particle dissolution plays a role in the differences between aerosol and conventional exposure.

### Intravenous exposure

Relevant exposure systems for injected NPs should mimic flow conditions. Several models indicated that contact of particles with the vessel wall occurs in a size-dependent manner resulting in particle-specific and flow-dependent optima of cellular uptake. The region near the surface of the epithelium, termed as lubrification plasma layer, is devoid of blood cells. Platelets and platelet-sized polystyrene particles of about 2 µm accumulate near the endothelium. This effect has been termed as margination and varies with particle material, size and shape. Margination of 100–500 nm functionalized polystyrene particles was significantly lower than that of the 2–5 µm large spheres. Wall deposition was higher for liposomes compared to gold and iron oxide NPs, for 65 nm liposomes higher than for 130 nm liposomes large particles, and for gold rods higher than for gold nanospheres [92]. Shear stress rates in the lubrification plasma layer are different from rates in the centre of the vessel. Values of 10–50 dyn/cm^2^ were calculated in the lubrification layer while mean wall shear stress in the centre of large arteries and veins is 2.7–4.5 dyn/cm^2^, ≤32 dyn/cm^2^ in small arteries and ≤11 dyn/cm^2^ in small veins [93].

Shear stress acted on particle parameters in different manner. When cells were cultured at 0.7, 3.0, 6.0 and 10.0 dyn/cm^2^ for 24 h and exposed to NPs for 60 min at these flow rates, 200 nm negatively charged methyacrylate-based NPs were best ingested at 10.0 dyn/cm^2^ [94]. For the positively charged particles, the inverse situation was observed. Differences in flow (0.1 and 0.5 dyn/cm^2^) versus static conditions were also reported for gold NPs by HUVEC [95]. While uptake at 0.5 dyn/cm^2^ was higher than at static condition, the uptake at 0.1 dyn/cm^2^ was lower than in the static condition. No cellular uptake was observed for 50 nm SiO_2_ NPs both in flow and static conditions, but uptake of CdTe NPs was higher at 0.5 dyn/cm^2^ than in static condition [96]. Based on the available studies, Cicha [97] concluded that no meaningful conclusions could be drawn because flow models, stress magnitudes and durations differed between the studies. When particle uptake by HUVEC was combined with cytotoxicity testing at different flow rates, the following effects were observed. Uptake of 2.7 and 4.7 nm CdTe NPs and 50 nm SiO_2_ NPs after 20 min was maximal at 0.5 dyn/cm^2^ and minimal in static condition [96]. Cytotoxicity determined at 24 h after exposure, on the other hand, was highest under static condition. Effects of adhering particles on the plasma membrane could explain cytotoxicity in the absence of cellular uptake.

A comparison of 10 different types of NPs (liposomes, lipid NPs, polymeric NPs, iron oxide NPs) showed that toxic effects on endothelial cells were lower in dynamic than in static condition. Culture in dynamic condition induced expression of endothelial phenotype and reduced cytotoxicity after 72 h from 100 µg/ml in static condition to 400 µg/ml in dynamic condition [98].

Coating of NPs with macromolecules from the biological environment (protein corona) has a marked effect on their cytotoxicity. Stimulating effects have been demonstrated on immune cells [99,100]. Despite the fact that the coating caused biological effects and many studies providing detailed characterization of the protein corona, NPs are usually applied in cell culture medium containing 10% foetal bovine serum and not coated with human plasma.

### Quantitative structure activity relationship

Conventional *in vitro* models have also been used to identify particle properties associated with adverse biological effects to assess risk by NP exposure and to optimize particles for medical application. The studies did not find such a correlation because particle varied in so many aspects that it was not possible to systematically vary one parameter leaving the others constant. The published data, however, were used to develop *in silico* models for risk assessment.

**Figure 3.**
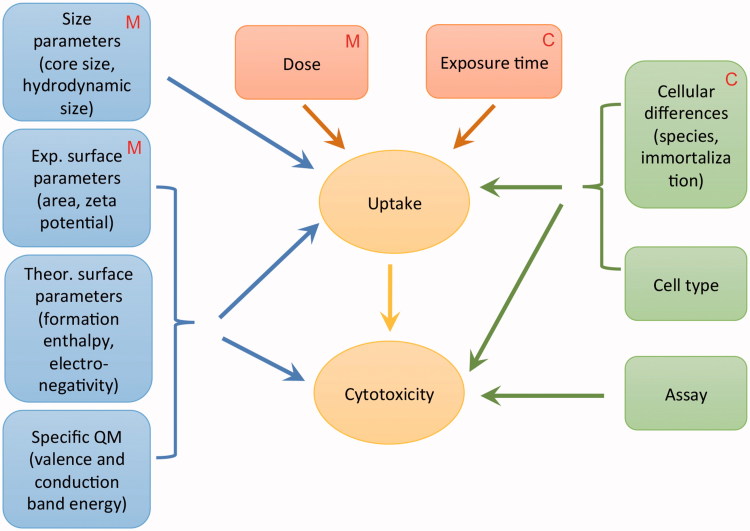
Particle and biological parameter that were identified to play a role in *in silico* modelling of metal oxide NPs (according to the meta-analysis by Ha et al. [105]). Parameters can be influenced by the use of advanced cell culture models, either by medium composition (M) or by the culture method (C). Medium composition may have an influence on aggregation (hydrodynamic size) and influence the dose that reaches the cell. In addition, surface parameters may be changed. The culture method influences mainly cellular differences by increasing cell differentiation and the exposure time as physiologically relevant culture methods usually enable exposure for longer time periods.

Quantitative structure activity relationship (QSAR) is routinely being used in the screening of compounds in drug development and in risk assessment of exposure to chemical entities. QSAR may provide an alternative for risk assessment of NPs to animal and *in vitro* studies, but particle parameters responsible for toxicity have not been clearly defined yet [101]. Nanotoxicologists agree that size is important. Other suggestions for relevant parameters are size distribution, surface area, surface chemistry, surface charge and surface porosity [102]. In addition to the above, purity, solubility, hydrophobicity and shape were suggested [103]. The OECD listed agglomeration, water solubility, zeta potential, octanol–water coefficient, size, surface area, porosity, surface chemistry, photocatalytic activity and ROS generation as relevant descriptors of NPs [104]. A recent meta-analysis of 216 articles identified 14 attributes contributing to the cytotoxicity of metal oxide particles [105]. These included experimental particle parameters (core size, hydrodynamic size, surface charge, specific surface area), general and specific quantum mechanic parameters (formation enthalpy, conduction band energy, valence bond energy, electronegativity) and biological parameters (assay, cell species, cell origin, cell type (normal/transformed)) in addition to dosage and exposure time (Figure 3). It may be assumed that dose and exposure time act mainly through particle uptake on cytotoxicity. Also size (primary size/aggregation) is estimated to act this way. Experimental and theoretical surface parameters and specific quantum mechanic parameters may influence cellular uptake in addition to directly causing cytotoxicity (e.g. by interaction with the plasma membrane). Advanced exposure models may cause some changes in the results by acting on specific parameters. The changed medium could influence agglomeration (hydrodynamic size) and change surface charge or/and reactivity. Since advanced culture methods usually lead to increased cell differentiation, cellular parameters, for instance particle uptake, may change. In addition, advanced culture will offer the possibility to expose cells longer to the NPs.

Current limitations for the establishment of good QSAR models include scarcity of high-quality studies that report complete particle characterization and use relevant exposure conditions [106]. The overview in Table 3 on QSAR models shows that variable numbers of parameters and types of descriptors (theoretical or experimental) have been used. One study included only one particle parameter [119], whereas other models were based on 30 descriptors [108]. It can be assumed that for the classification of similar particles, a lower number of descriptors may be needed. It is also important how directly the predicted readout is linked to a particle property. Energy band structure of metal oxide NPs may have a relatively direct influence on particle reactivity and oxidative stress. Some descriptors, on the other hand, did not appear to have any effect on cytotoxicity. The model by Wang et al. based on 18 NPs with seven cellular assays and seven particle parameters (Table 3) identified zinc and cadmium content, radical activity, surface area and reactivity as risk factors for cytotoxicity [107]. Conduction band energy and ionic index out of a panel of 30 theoretical descriptors was identified as very predictive for cytotoxicity of metal oxide particles [108]. By contrast, another study reported that no single particle parameter but only the combination of primary size, spin–lattice, spin–spin relaxivities, zeta potential could classify iron oxide particles regarding their cytotoxicity [109]. *In silico* testing has the advantage that different models can be compared. Re-analysis of data from Zhou et al. (2008) and Shaw et al. (2008) with another model produced similarly good results [110].

**Table 3.** Parameters included in QSAR models.

Nanomaterial	Toxicity endpoint	Characterization	Reference
18 NMs (carbon-based, metal oxides)	Cytotoxicity, apoptosis, pro-inflammatory effects, haemolysis, viability, mitochondrial membrane potential, morphology	7 descriptors: size, surface area, morphology, metal content, reactivity, free radical generation, zeta potential	[107]
18 NMs	Viability	17 quantum mechanical descriptors (enthalpy of formation of nanocluster, total and electronic energy, core–core repulsion energy, solvent accessible surface, energy of the highest occupied molecular orbital, energy of the lowest unoccupied molecular orbital, gap between both, electronic chemical potential, valence band, conduction band, Mulliken’s electronegativity, Parr and Pople’s absolute hardness, Schüürmann Molecular Orbital shift alpha quantities, polarizability derived from the heat of formation, and polarizability derived from dipole moment) and 11 experimental descriptors (area, volume, surface diameter, volume/mass diameter, volume/surface diameter, aspect ratio, porosity, sphericity, circularity)	[111]
51 NMs with four metal core structures	Viability, reducing equivalents, apoptosis, mitochondrial membrane potential	5 descriptors: core composition, coating, surface modification, relaxivities, zeta potential	[112]
42 NMs with two cores	Cytotoxicity	6 descriptors: primary particle size, size in water, size in PBS, cell in cell culture medium, concentration, zeta potential	[113]
13 pure, core-shell and alloy Au/Pd TiO_2_ NMs	Cytotoxicity (CHO-K1 cells)	2 descriptors: size, surface area	[114]
9 metal oxide NMs	Cytotoxicity (BEAS-2B cells)	14 descriptors: atomization energy of the metal oxide, period of the NP metal, and NP primary size, in addition to NP volume fraction (in solution) were identified as most predictive	[115]
24 metal oxide NMs	ROS, oxidative stress, pulmonary inflammation in mice	30 theoretical descriptors: conduction band energy predictive for some, solubility for other metal oxide NPs	[116]
41 metal oxide NMs	Cytotoxicity	4 descriptors; size, electronegativity, polarizability, molar volume	[117]
17 metal oxide NMs	Cytotoxicity (HaCaT cells)	7 theoretical descriptors (number of metal atoms, number of oxygen atoms, molecular weight, charge of the metal cation corresponding to a given oxide, metal electronegativity, sum of metal electronegativity for the individual metal oxide, sum of metal electronegativity for the individual metal oxide divided by the number of oxygen atoms in a specific metal oxide)	[118]
24 metal oxide NMs	Viability, 2 cell lines	30 descriptors: conduction band energy and ionic index were identified as very predictive	[108]
44 iron oxide NMs	4 cell types, 4 assays	4 descriptors: primary size, spin–lattice, spin–spin relaxivities, zeta potential; no single parameter performed best	[109]
6 metal oxide NMs	Oxidative stress	1 descriptor: energy band structure	[119]
307 studies, Cd quantum dots	Viability	24 qualitative and quantitative features (ligand, shell, surface modification, assay type, exposure time, exposure concentration, cell anatomical type, cell origin)	[120]
20 C60 fullerene NPs	Mutagenicity	3 descriptors: dose, illumination, metabolic activation	[121]
84 f-MWCNTs	Cytotoxicity, protein binding, immune response	5 descriptors: zeta potential, electrophoretic mobility, surface area, porosity, solubility	[122]

## Conclusion

Physiologically relevant (advanced) *in vitro* systems can improve the physiological relevance of routine cell culture. This makes them useful tools for the mechanistic understanding of NP toxicity. The possibility to assess the effect of cell multilayers, mucus and of cellular interaction on particle effects as well as the possibility of relevant exposure to aerosols, particles in flow condition and suspension in simulated body fluids are the main advantages compared to conventional culture. Depending on the type of NPs and the cellular models, the observed effects differed between conventional and advanced culture systems. Cytotoxicity was usually lower in 3D than in conventional culture, but 3D models were often more sensitive to identify cellular reactions to NPs [68]. Limitations of advanced exposure systems are the introduction of artificial barriers (scaffolds, membranes) and adhesion of particles to parts of the exposure systems. Advanced culture systems are more expensive, technically more demanding, more difficult to standardize and usually less suitable for HTS. It is currently not clear to which extent the advanced culture systems provide more predictive data for toxicity in humans than the conventional systems. Validation using human data is usually not possible because such data are rare. Generation of animal data is ethically and financially problematic and the predictive value limited due to potential species-specific differences. Databases containing results from conventional cell culture, advanced models and animal experiments, however, could be useful to determine the role of advanced culture systems in the toxicological assessment of NPs. Furthermore, they are useful for the establishment of predictive QSAR models.

## Supplementary Material

Table_S1.docx
