# Supplementary material for: Comparison of conventional and advanced in vitro models in the toxicity testing of nanoparticles
Source: Artif Cells Nanomed Biotechnol. 2018 Jun 29;46(Suppl 2):1091–107. doi: 10.1080/21691401.2018.1479709 (PMC6214528; doi:10.1080/21691401.2018.1479709)
Supplement: Table_S1.docx [file IANB_A_1479709_SM4646.docx]

Table S1: Cell composition of commercially available reconstructed tissues

| Company | Product | Cell composition |
| --- | --- | --- |
| Atera | Reconstructed Human Conjunctival Epithelium | Chang cells |
|  | Reconstructed Human Full-Thickness (RHFT) | Epidermal keratinocytes + melanocytes + fibroblasts |
| Cyprotex | Cardiotoxicity assay | Human iPS cell-derived iCell® cardiomyocytes |
|  | Hepatotoxicity assay | 3D HepaRG cells |
| EpiCS® | epiCS (epidermis) | Epidermal keratinocytes |
|  | epiCS-M (pigmented epidermis) | Epidermal keratinocytes + melanocytes |
| Epithelix | MucilAir™ (bronchial epithelium) | Ciliated + goblet + basal cells |
|  | MucilAir™-HF (bronchial epithelium with fibroblasts) | Ciliated + goblet + basal cells + fibroblasts |
|  | SmallAir™ (bronchiolar epithelium) | Ciliated + goblet + club + basal cells |
|  | SmallAir™-HF (bronchiolar epithelium with fibroblasts) | Ciliated + goblet + club + basal cells + fibroblasts |
|  | OncoCilAir™ (bronchial tumor) | Bronchial epithelial + tumor cells + fibroblasts |
| Insphero | 3D InSight™ Custom Microtissues | Epithelial + endothelial + immune cells |
|  | 3D InSight™ Liver | Hepatocytes +/- Kupffer cells^1^ |
|  | 3D InSight™ Pancreatic microislets | Insulin, glucagon, somatostatin secreting cells |
|  | 3D InSight™ Tumor | Cancer cell lines + fibroblasts ± human umbilical vein endothelial cells |
| Japan tissues engineering Inc. | LabCyte Cornea-Model | Corneal epithelial cells |
|  | LabCyte Epi-Model | Epidermal keratinocytes |
| SkinEthic | SkinEthic™-HCE (Corneal epithelium) | Corneal epithelial cells |
|  | SkinEthic™-RHE, EpiSkin™ (Epidermis) | Epidermal keratinocytes |
|  | T-Skin™ | Epidermal keratinocytes + fibroblasts |
|  | SkinEthic™-HGE (Gingival epithelium) | Gingival epithelial cells |
|  | SkinEthic™-HOE (Oral epithelium) | Squamous cell carcinoma cells |
|  | SkinEthic™-RHPE (Pigmented Epidermis) | Epidermal keratinocytes + melanocytes |
|  | SkinEthic-HVE (Vaginal epithelium) | Vulval epidermoid carcinoma cells |
| Mattek Coop. | EpiAirway (bronchial epithelium) | Bronchial epithelial + basal + goblet cells |
|  | EpiAirwayFT (bronchial epithelium with fibroblasts) | Bronchial epithelial + basal + goblet cells + fibroblasts |
|  | EpiAlveolar (alveolar epithelial cells) | Alveolar epithelial + endothelial cells |
|  | EpiCorneal (Cornea) | Corneal epithelial cells + fibroblasts |
|  | EpiDerm (Epithelium) | Epidermal keratinocytes |
|  | EpiDermFT (epidermis with fibroblasts) | Epidermal keratinocytes + melanocytes |
|  | EpiGingival (Gingival epithelium) | Oral epithelial cells |
|  | EpiIntestinal (intestinal epithelium) | Enterocytes + Paneth^2^ + M + tuft^3^ + intestinal stem cells |
|  | EpiIntestinalFT (intestinal epithelium with fibroblasts) | Enterocytes + Paneth + M + tuft + intestinal stem cells + fibroblasts |
|  | EpiOcular (corneal epithelium) | Corneal epithelial cells |
|  | EpiOral (oral epithelium) | Oral epithelial cells |
|  | EpiVaginal (vaginal epithelium) | Vaginal epithelial + dendritic cells |
|  | EpiVaginalFT (vaginal epithelium) | Vaginal epithelial + dendritic cells + fibroblasts |
|  | MelanoDerm (Epithelium) | epidermal keratinocytes + melanocytes |
|  | Melanoma (full thickness melanoma) | Epidermal keratinocytes + melanoma cells + fibroblasts |
|  | Psoriasis (Epithelium with fibroblasts) | Epidermal keratinocytes + psoriatic fibroblasts |
| Organovo | Custom 3D tissue models (bioprinted) | All cells of the original tissue |
|  | exVive™ 3D Liver Tissue | Hepatocytes + hepatic stellate^4^ + endothelial + Kupffer cells |
|  | ExVive™ Human Kidney Tissue | Proximal tubule epithelial cells + renal fibroblasts + endothelial cells |
| Stemcell Technologies | PneumoCult™ Ex, PneumoCult™ ALI | Bronchial epithelial cells in submersed or air-liquid interface condition |
| zenbio | ZenSkin RHE | Epidermal keratinocytes |

^1^ macrophages of the liver

^2^ chemosensory cells in the epithelial lining of the intestines and respiratory tract

^3^ secretion of defensins, lysozyme and tumor necrosis factor alpha

^4^ vitamin A-and lipid storing cells in the liver, also called Ito cells
